# Development of Primary Monolayer Cell Model and Organotypic Model of Uterine Leiomyoma

**DOI:** 10.3390/mps5010016

**Published:** 2022-02-06

**Authors:** Natalia Shved, Anna Egorova, Natalia Osinovskaya, Anton Kiselev

**Affiliations:** Department of Genomic Medicine, D.O. Ott Research Institute of Obstetrics, Gynecology and Reproductology, MendeleevskayaLine 3, 199034 Saint-Petersburg, Russia; natashved@mail.ru (N.S.); egorova_anna@yahoo.com (A.E.); natosinovskaya@mail.ru (N.O.)

**Keywords:** uterine leiomyoma, cell model, organotypic model, *MED12*, *HMGA2*

## Abstract

Cellular technologies are one of the most promising areas of biomedicine, which is based on the isolation of cells of various types, followed by their cultivation and use, or the use of their metabolic products, for medical purposes. Today, a significant part of biomedical research is carried out in vitro. On the other hand, organotypic culture can be used as a powerful model system and can complement cell culture and in vivo studies in different biomedical applications. Uterine leiomyoma (UL) is a very common benign tumor and often leads to many reproductive complications. Herein we describe a fast and reliable method of isolation and UL primary cells culturing along with the development of a UL organotypic model. We propose the usage of UL primary cells in experimental work at a first passage to prevent loss of driver mutations in *MED12* and *HMGA2* genes. New optimized conditions for the growth and maintenance of 2D and 3D models of uterine leiomyoma in vitro are suggested.

## 1. Introduction

Uterine leiomyomas (UL) are the most common benign tumors and represent a major public health problem in women’s reproductive health. The factors affecting their growth are of great interest and are often studied in vitro using cellular models, which makes this approach a widespread model system for studying the pathogenesis and drug therapy of the disease [1,2,3]. Modern data convincingly indicate that somatic mutations of *MED12* and rearrangements involving the *HMGA2* gene are the most frequent driver mutations in UL [4]. Furthermore, new types of chromosomal deletions and translocations often appear in the myomatous nodes. Among them, the most common are translocations that occupy the chromosome region 12q14–15 and deletions of a part of the long arm of chromosome 7 [4]. A number of studies have shown that mutation inthe *MED12* gene coincides with a severely reduced ability of UL cells to grow in vitro, which leads to their rapid disappearance in primary cell culture and casts doubt on the usefulness of existent UL cellular models for biomedical research [5,6,7].To date, several techniques have been described for the isolation of UL cell cultures but all of them cannot preserve *MED12* gene mutations [8,9]. Primary cultures derived from UL nodes, presumably of non-tumor origin, as well as tumors carrying *HMGA2* rearrangements, can survive much longer [5,6].

UL is a benign tumor consisting of various cell populations (fibroblasts, smooth muscle, and stem cells) embedded in the extracellular matrix, interacting with each other, thereby ensuring proliferation. Organotypic cultures are used to study the pathogenesis and various therapeutic approaches for a number of malignant and benign tumors [10]. They retain a histological and three-dimensional structure with inter- and extracellular interactions, cellular matrix components, and intact metabolic activity.

The aim of the present study is to developa technique for preparinga short-term monolayer cell culture and 3D organotypic model from UL tissue obtained after myomectomy or hysterectomysurgery.

## 2. Experimental Design

**Figure 1:**
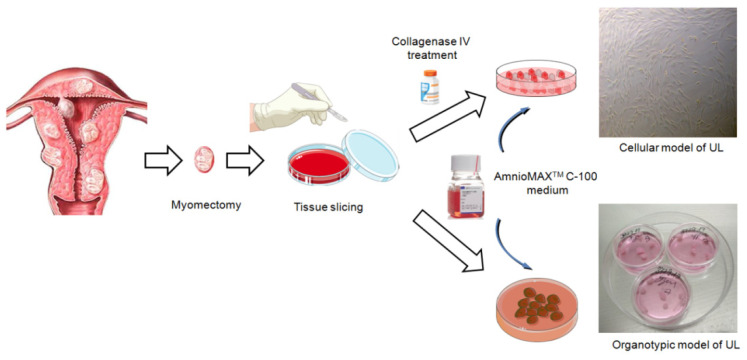
Representative scheme of UL cellular and organotypic model development.

We have designed a protocol for the development of cellular and organotypic models from uterine fibroids obtained after myomectomy/hysterectomy. In our protocol, we describe step by step development of the models as summarized in Figure 1. This protocol will be very useful for researchers who study molecular mechanisms of UL pathogenesis and genetic alterations, and develop various therapeutic approaches. The biomaterial for the model development is dissected myomatous nodes obtained after myomectomy. The tissue slicing is performed under sterile conditions. All instruments are sterilized by autoclaving before the procedure. Cellular model development requires collagenase IV treatment to destroy UL tissue and ensure the migration of UL cells from the dissected UL fragments. In the case of an organotypic model, no enzymatic treatment is needed and bigger UL fragments (3 × 3 × 3 mm) should be left intact. The most critical component is Gibco^®^AmnioMAX™ C-100 Complete Medium, which must be used to ensure the viability of UL cells and tissues in culture. The obtained UL cell and organotypic cultures can be tested for the presence of main driver genetic alterations – mutations in exon 2 of the *MED12* gene and overexpression of the *HMGA2* gene. In fact, the genetic analysis should be considered as the main parameter for characterization of the obtained models.

### 2.1. Materials

- Serological pipettes (Pasteur, 3 mL, PE, PU, sterile, graduated) (Biologix, Shandong, China; Cat. no.: 30-0138A1)
- Culture bottle “T-25”, for work with adhesive cell cultures (TC treated), lid with filter, sterile (Corning; Wiesbaden, Germany; Cat. no.: Corning-3815)
- Plastic sterile containers, volume 120 mL(Medpolymer, Saint-Petersburg, Russia; Cat. no.: 2620304)
- Sterile plastic Petri dishes, 94 × 16 mm (Greiner, Frickenhausen, Germany; Cat. no.: Greiner-633181)
- Sterile plastic Petri dishes, 35 mm, for working with adherent cell cultures, ventilated (Corning, Wiesbaden, Germany; Cat. no.: Corning-353001)
- 15 mL volume centrifuge tubes (Corning, Wiesbaden, Germany; Cat. no.: 430053)
- 1.7 mL volume centrifuge tubes (Corning, Wiesbaden, Germany; Cat. no.: 3207)
- 2.0 mL volume centrifuge tubes (Corning, Wiesbaden, Germany; Cat. no.: 3213)
- PCR tubes 0.5 µL (SSI, Lodi, CA, USA, Cat. no.: SSI-3320-00)
- PCR tubes 0.2 µL (SSI, Lodi, CA, USA, Cat. no.: SSI-3225-00)
- Tips for automatic pipettes 200 µL, 1000 µL, 5000 µL (Axygen, Union, CA, USA; Cat. no.: TF-200-R-S, T-1000-B-R-S, T-5000-C)
- Surgical scissors, sterile (Surgicon, Punjab, Pakistan; Cat. no.: 1269184)
- Sharp-tipped metal J-16-140, sterile (Surgicon, Punjab, Pakistan; Cat. no.: 1269194)
- Cryotubes, sterile (Deltalab S.L., Barcelona, Spain; Cat. no.: 409106.1)
- PCR plates; 96-well; ABI-compatible (SSI, USA, Cat. no.: SSI-3425-00)
- Gibco^®^AmnioMAX™ C-100 Complete Medium (Thermo Fisher Scientific, Waltham, MA, USA; Cat. no.: 12558011)
- Gibco^®^AmnioMAX™ C-100 Basal Medium (Thermo Fisher Scientific, Waltham, MA, USA; Cat. no.: 17001074)
- Gibco^®^AmnioMAX™ C-100 Supplement (Thermo Fisher Scientific, Waltham, MA, USA; Cat. no.: 12556023)
- Eagle’s MEM nutrient medium (BioloT, SaintPetersburg, Russia; Cat. no.: 1.3.3.)
- Collagenase type IV (*Clostridium histolyticum*), 200 U/mL(Sigma Aldrich, Steinheim, Germany; Cat. no.: Si C5138-1G)
- 1 × 0.25% trypsin solution (BioloT, SaintPetersburg, Russia; Cat. no.: 1.2.2.5.)
- 0.3% Versen’s solution (BioloT, SaintPetersburg, Russia; Cat. no.: 1.2.3.2.)
- Sterile saline solution 0.9% NaCl (BioFarmGarant, Vladimir, Russia; Cat. no.: 322664)
- DPBS without Ca and Mg (SigmaAldrich, Steinheim, Germany; Cat. no.: 59331C-1000ML)
- 96° ethyl alcohol P.O.A. (Merck, Gernsheim, Germany; Cat. no.: 8.18760.1000)
- Penicillin-Streptomycin Solution 10.000 IU/mL (Gibco, ThermoFisher Scientific, Waltham, MA, USA; Cat. no.: 15140122)
- Kit for reverse transcription with MMLV-RH (DiAM, Moscow, Russia; Cat. no.: 1967.0050)
- RT-PCR Kit with EVA Green dye (Syntol, Moscow, Russia; Cat. no.: R-441)
- Fetal bovine serum (BioloT, SaintPetersburg, Russia; Cat. no.: 1.1.10.7.)
- Dimethylsulfoxide (DMSO) (VWR (Amresco), Mont-Royal, QC, Canada; Cat. no.: Am-0231-0.1)
- RNAlater^TM^ solution (Invitrogen, Thermo Fisher Scientific, Waltham, MA, USA; Cat. no.: Am-7020)
- Big Dye Terminator v.3.1 Kit (Applied Biosystems, Troy, NY, USA; Cat. no.:4337455)
- Big Dye XTerminator Purification Kit (Applied Biosystems, Troy, NY, USA; Cat. no.: 4376487)
- Kit for reverse transcription (Syntol, Moscow, Russia; Cat. no.: OT-1)
- Mixture of dNTP, 10 mM (10 µmol) each (Syntol, Moscow, Russia; Cat. no.: N1103)
- Taq polymerase, buffer without Mg^2+^ and 25 mM MgCl_2_ (Syntol, Moscow, Russia; Cat. no.: E0120)

### 2.2. Equipment

- Set of automatic pipettes volume 10–100 µL, 100–1000 µL, 1000–5000 µL (Sartorius BIOHIT, Göttingen, Germany; Cat. no.: 725050,725070, 725080)
- Biosafety Cabinet Class II (Laminar systems, Miass, Russia; Cat. no.: 1R-D.001-12ada)
- Ultraviolet germicidal irradiator (recirculator) Desar (Himmedservis, Tver, Russia; Cat. no.: av345)
- Centrifuge for 15 mL tubes up to 2300× *g* (ELMI Ltd., Riga, Latvia; Cat. no.: Elmi CM-6M)
- Centrifuge MiniSpin (Eppendorf, Hamburg, Germany; Cat. no.: 00000030762)
- CO_2_ incubator MCO-19AIC(UV)(SANYO Electr. Co., Osaka, Japan; Cat. no.: SA-MCO19)
- Incubator +37 °C (Memmert, Germany; Cat. no.: 9537930)
- Two-compartment refrigerator: +40 °C and −20 °C (POZIS, Zelenodolsk, Tatarstan, Russia; Cat. no.: 00000031036)
- Refrigerator −80 °C (SANYO Electr. Co., Osaka, Japan; Cat. no.: MDF-U32V)
- Inverted microscope MIBR with a digital camera (LOMO, Saint Petersburg, Russia; Cat. no.: 00000074356)
- Centrifuge mini-vortex microspin (BIOSAN, Riga, Latvia; Cat. no.: 00000026197)
- Thermocycler Rotor-Gene 3000 (Corbett Research, Mortlake, NSW, Australia)
- Laboratory roller mixer-rotator (BIOSAN, Riga, Latvia; Cat. no.: BS-010133-AAG)
- Capillary genetic analyzer (Sanger DNA sequencer) (Applied Biosystems, Carlsbad, CA, USA; Cat. no.: A28978)
- Thermocycler T100 (BIO-RAD, Hercules, CA, USA; Cat. no.: 00010014822)

## 3. Procedure

### 3.1. Obtaining Primary Cell Culture from Fragments of Collagenase-Treated Leiomyoma Nodules


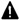
**CRITICAL STEP** One hour before handling the material, turn on the Desar and UV lamp in the Biosafety Cabinet Class II. Ensure that all the procedures are completed under sterile conditions.

**Figure 2:**
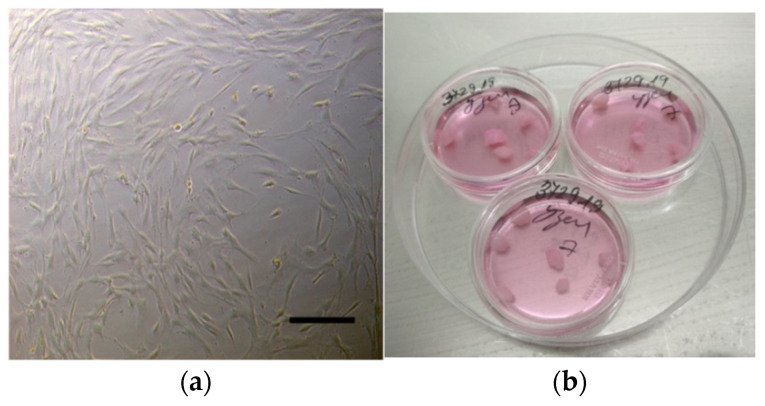
Representative microphotographs of the cellular and organotypic UL models: (**a**) primary UL cells at p0 (magnification × 100; scale bar is 100 µm); (**b**) typical appearance of leiomyoma fragments.

1. Wash tumor fragments, total volume not less than 3.0–3.5 cm^3^, in three changes of saline, then place in a Petri dish with 15 mL of saline and shred into fragments about 3 × 3 × 3 mm in diameter using a scalpel (scissors).
2. Then, transfer the tissue fragments to 15 mL centrifuge tubes with 5 mL of Eagle’s MEM nutrient medium (Biolot, Saint-Petersburg, Russia) and 2.5 mg of type IV collagenase (Sigma Aldrich, Saint Louis, MO, USA), which corresponds to enzyme activity of 200 U/mL.
3. Incubate tubes with tumor fragments in a tilted position for 90–120 min at 37 °C, shake or resuspend periodically.
4. After incubation, add 5 mL of DPBS to the tubes and resuspend the tissue fragments using a 5-mL automatic pipette to obtain a cell suspension.
5. Then, centrifuge the cells for 10 min at 200× *g*, remove the supernatant, and separate the precipitate.
6. Then, add 5 mL of DPBS to the tubes again, well resuspend the precipitate, and centrifuge for 10 min at 200× *g*, remove the supernatant, and resuspend the precipitate again.
7. Then, add 2 mL of pre-diluted AmnioMax Basal Medium with AmnioMax Supplement serum to the tubes. Transfer the resulting suspension to culture flasks T-25 (Thermo Fisher Scientific, Waltham, MA, USA) using a disposable sterile Pasteur pipette and incubate for 12–18 days at 37 °C and 5% CO2 to reach 80% confluence. Then the culture medium should be changed every 3–4 days.
8. When primary (p0) UL cultures reach 80% confluence (Figure 2a), they can be used for experiments or for the first passaging. We recommend taking at least 10–15% of p0 cells to achieve 80% confluence of p1 culture in 7–12 days. The viability of cells after passage can be monitored by the Trypan Blue exclusion test.

### 3.2. Freezing of Leiomyoma Cell Cultures and Nucleic Acid Isolation


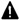
**CRITICAL STEP** The following protocol steps should be performed in the Biosafety Cabinet Class II.

1. Wash the cells twice with Versen’s solution.
2. Remove the cells by adding 700 µL of Trypsin-Versen solution to each well (1:3 ratio).
3. Incubate for 4–5 min at +37 °C. 4.
4. Add 5.0 mL of 0.9% NaCl and gently wash cells from the bottom of the vial.
5. Transfer the cell suspension to 15.0 mL centrifuge tubes.
6. Then, centrifuge the cells for 10 min at 200× *g*, remove the supernatant, and separate the precipitate.
7. Then, add 5 mL of DPBS to the tubes again, well resuspend the precipitate, and centrifuge for 10 min at 200× *g*, then remove the supernatant.
8. Add 300 μLof DPBS solution without Ca and Mg, mix gently.
9. Transfer 100 µL of the cell suspension to a 2.0 mL tube containing 1.0 mL of RNA stabilizer. Leave at room temperature for 1 h. Then use for RNA isolation or store in a −80 °C freezer for up to 1 year if necessary.
10. Transfer 100 µL of cell suspension to a 2.0 mL dry tube and use for DNA extraction. If necessary, store in a −80 °C freezer for up to 1 year.
11. Transfer 100 µL of cell suspension to a 2.0 mL cryotube containing a mixture of 100 µL dimethyl sulfoxide and 900 µL bovine serum. Mix gently by pipetting. Cryotubes should bescrewed thoroughly. Label cryovials with a water- and low-temperature resistant marker. Label cryoprobes with the date, culture name, and passage. If necessary, store in a freezer at −80 °C for no more than 1 year. According to our observations, when p0 leiomyoma cell lines are thawed, cells carrying the *MED12* mutation and/or increased expression of the *HMGA2* gene are preserved in p1 culture.

### 3.3. Organotypic Model from Native Fragments of Leiomyoma Nodules

1. Using scissors or a scalpel, divide a large fragment of the node into smaller ones (approximately 3 × 3 × 3 or 4 × 4 × 4 mm) in the number necessary for subsequent experiments.
2. Transfer 5–7 fragments to each 35 mm diameter Petri dish (Figure 2b).
3. Add 1.0 mL of Gibco^®^AmnioMAX™ C-100 Complete Medium. Place in a CO_2_ incubator. According to literature data and personal observations, cells in tumor slices remain viable at least for 7–21 days [10]. It is recommended to keep tumor fragments ex vivo in culture for up to 7 days with the medium change every 3–4 days when using them in experimental work.

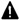
**CRITICAL STEP** The culture medium should not completely cover the fragments, providing contact with air (10–15% of the fragment surface) in the CO_2_ incubator and preventing it from floating up. According to our observations, on the 5–7th day of cultivation, the cells begin to migrate into the culture medium and ensure the fixation of the UL fragment on the substrate.

### 3.4. MED12 Mutation Analysis

Analysis of *MED12* exon 2 mutations was performed as described previously by direct PCR sequencing of genomic DNA according to the protocol repeatedly used in our laboratory [11,12].

### 3.5. Reverse Transcription of RNA and HMGA2 Gene Expression Analysis

#### 3.5.1. Reverse Transcription of RNA

Reverse transcription of RNA was performed using the kit for reverse transcription “OT-1” (Syntol, Moscow, Russia) according to the manufacturer’s instructions. The reverse transcription kit is based on recombinant reverse transcriptase, which is a product of the pol gene of Moloney Murine Leukemia Virus (M-MLV).

1. Perform eachreaction in a total volume of 20 μL containing 10 mmol d-NTP and 200 U/mL MMLUV in the presence of oligo-dT16 (2.5 mM) and random hexamers (3 mg/mL) with the addition of 100 to 500 ng total RNA.
2. Place strips of random oligonucleotide hexamers with the reaction mixture into Thermocycler T100. The reverse transcription reaction was performed at: +25 °C–10 min; +37 °C–120 min; +85 °C–5 min; +4 °C–5 min. The cDNA was stored at +4 °C. The obtained cDNA was used for real-time PCR.

#### 3.5.2. HMGA2 Gene Expression Analysis

Calibration curve preparation for Real-Time PCR

1. 12 µL cDNA mixture [x ng/mkl] + 12 µL H_2_O, stir by pipetting, incubate for 10 min.
2. 12 µL of [x/2 ng/mkl] cDNA mixture + 12 µL H_2_O, stir by pipetting, incubate for 10 min.
3. 12 µL of [x/4 ng/mkl] cDNA mixture + 12 µL H_2_O, stir by pipetting, incubate for 10 min.
4. 12 µL of [x/8 ng/mkl] cDNA mixture + 12 µL H_2_O, stir by pipetting, incubate for 10 min.
5. 12 µL of [x/16 ng/mkl] cDNA mixture + 12 µL H_2_O, stir by pipetting, incubate for 10 min.
6. The calibration dilutions must be stored at −20 °C.

Real-Time PCR.


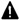
**CRITICAL STEP** The following steps of the protocol are performed in the PCR box.

1. Take out the strips and the lids for the strips using tweezers.
2. In 1.5 mL tubes (or 2 mL-depending on the number of samples), make separate mixtures for each set of primers at a concentration of 5 pmol (*HMGA2* gene: forward 5′-AGA GTC CCT CTA AAG CAG CTC A-3′; reverse 5′-CAA CTG CTGCTG AGG TAG AAA TCG-3′), including primers to the housekeeping gene (β-actingene:5′-TGC CGA CAG GAT GCA GAA G-3′; reverse 5′-GCC GAT CCACAC GGA GTA CT-3′). Tubes for the mixtures should be wrapped in foil.
3. Scheme of mixture preparation (per sample taking into account prepared standards and one blank sample; in order of adding reagents):
  µL H_2_O
  4 µL MgCl_2_ (25 mM)
  µL dNTP’s (2.5 mM)
  2.5 µL EvaGreen (10×)
  1 µL F-primer
  1 µL R-primer
  0.3 µL Taq-pol.
4. Stir the mixture by pipetting; pipette into 22.5 µL strips. Try to dig close to the bottom of the strips; do not leave any droplets on the walls at the top.
5. Using separate spouts, dig 2.5 µL of cDNA into strips. Drop each sample in two replicates.
6. Close the test tubes with caps. Place in the amplifier Rotor-Gene 3000 (Corbett, Australia) and run the appropriate program (Single cycle 95°–5 min; then 38 cycle: 94°–15 s; 600–60 s. Then heating from 60° to 95° in 0.5 degree increments, 10 s each step).
7. The relative level of mRNA expression was calculated by the ΔΔCt method (Livak method) using ExpressionSuit V1.0.3 software.

## 4. Expected Results

Primary cell and tissue fragments taken directly from the body represent the most accurate models for in vitro studies. In the case of uterine leiomyoma, despite monoclonal origin, various cell populations can be found, including smooth muscle, fibroblasts, and stem cells, embedded in an abundant extracellular matrix [13,14]. The communication between these cells is likely critical for UL proliferation and survival. At the same time, the rapid loss of mutated cells in the monolayer model does not affect in vitro growth as a result of as yet unknown mechanisms and indicates the need for soluble factors or components that are absent in the standard culture medium.

We propose using the complete Gibco^®^AmnioMAX^TM^ C-100 medium specially designed to support the growth of various cell types to ensure the survival of monolayer primary cell culture and 3-dimensional organotypic model. It should be noted that we tested different media at the beginning of the protocol development (Figure S1). According to our observations, fibroblast-like cells migrating into the culture medium from fragments of myomatous nodes give rise to the first colonies, and they preserve a mutation in the *MED12* gene for at least three weeks of culturing (Figure 2). It should be noted that the cells had good viability after passaging as it was determined by the Trypan Blue exclusion test; no more than 5% of cells were Trypan Blue-positive.

**Figure 3:**
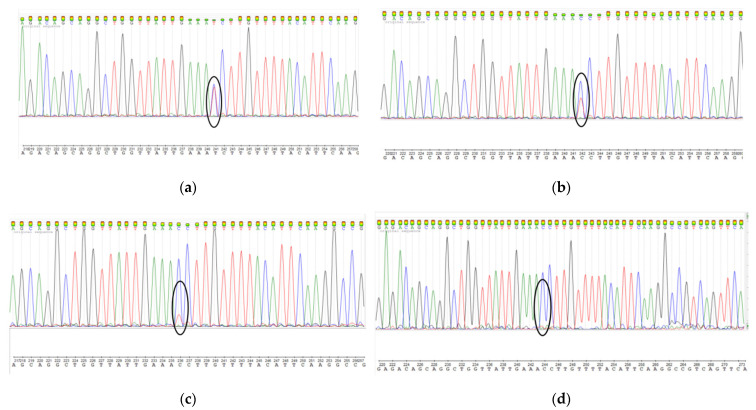
Detection of heterozygous mutation c.131G >A in *MED12* gene in the same primary UL cell line at different passages: (**a**) p1; (**b**) p2; (**c**) p3; (**d**) control–healthy myometrium.

In order to minimize the loss of the cells carrying a driver mutation in the *MED12* gene, we propose to use only the p1 subculture. Thus, we recommend increasing the UL tissue volume to obtain cell culture at the first passage (p0). The mutation preservation was confirmed by the screening of *MED12* exon 2 mutations using Sanger sequencing (Figure 3).

**Figure 4:**
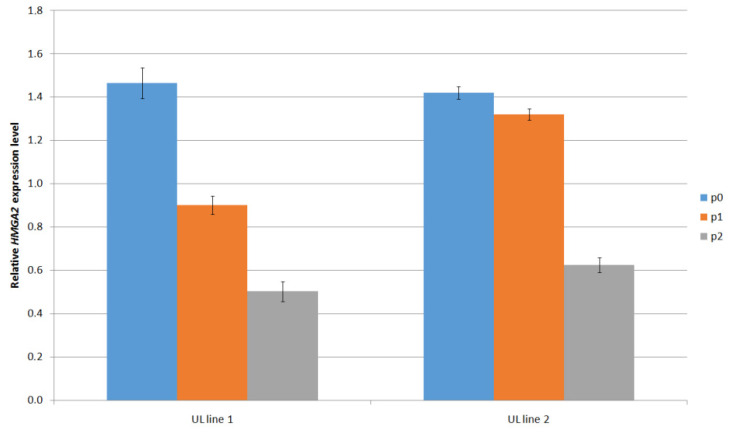
Measurement of *HMGA2* gene expression (fold change relative to reference β-actin gene) in two independent primary UL cell lines at different passages.

The presence of *HMGA2* gene expression, another genetic alteration in UL was confirmed by the RT-qPCR method (Figure 4). It can be seen that the *HMGA2* gene expression level significantly decreases with the higher passage.

Thus, we recommend using at least 30 mm^3^ volume of initial tissue before starting the isolation of UL cells. After the collagenase treatment and subsequent cell culturing during 1–2 weeks, 2–3 flasks with 80% confluency (0.8–1.5 × 10^6^ UL cells) can be obtained. It is worth noting that due to individual heterogeneity between leiomyomas, there may be a difference in proliferation rate per each UL line. According to our observations, most UL lines doubled every 24 h. The obtained UL lines at p0 can be frozen or used directly for experimental work e.g., seeded at 24- or 48-well plates at p1. 

In the case of the organotypic model, we found that the *MED12* gene mutations and *HMGA2* gene expression–two main drivers of UL pathogenesis–remain preserved for at least 14 days of culturing. For viability evaluation, the UL fragments were transferred to a new Petri dish on days 7, 14, and 21 to obtain cultures of cells that migrated from the UL fragments. The viability of migrated cells was monitored by the Trypan Blue exclusion test. The percentage of Trypan Blue-positive cells did not exceed 5%. All the cell lines obtained in this way had the same mutations in the *MED12* gene as a parent UL fragment.

**Figure 5:**
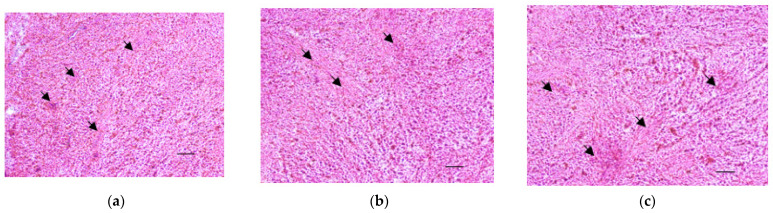
Histological characterization of the organotypic model. Hematoxyline-eosine stained cryosections of UL fragments after 7 (**a**), 14 (**b**), and 21 (**c**) days of culture (magnification × 100; scale bar is 200 µm). Arrows mark extracellular matrix.

It should be noted that cultured UL fragments retain their morphology as it was assessed by hematoxyline-eosin stainingon days 7, 14, and 21 (Figure 5 and Figure S2). In the stained sections, a densely packed, disorganized pattern of extracellular matrix (marked by arrows) and fibrous connecting tissue can be seen.

## Data Availability

The data presented in this study are available on request from the corresponding author. The data are not publicly available due to restrictions of the subjects’ agreement.

## Supplementary Materials

The following supporting information can be downloaded at: https://www.mdpi.com/article/10.3390/mps5010016/s1, Figure S1: Detection of heterozygous mutation c.131G˃A in MED12 gene in the same primary UL cell line at passages p0 and p1 cultured with AmnioMAX™ C-100 or DMEM F-12 medium; Figure S2: Histological characterization of the organotypic model. Hematoxyline-eosine stained cryosections of UL fragments after 7, 14 and 21days of culture (magnification × 200).

## References

[1] Ohara N. (2008). Action of progesterone receptor modulators on uterine leiomyomas. Clin. Exp. Obstet. Gynecol..

[2] Zhu Y., Zhang T., Xie S., Tu R., Cao Y., Guo X., Zhou J., Zhou X., Cao L. (2012). Gestrinone inhibits growth of human uterine leiomyoma may relate to activity regulation of ERα, Src and P38 MAPK. Biomed. Pharmacother..

[3] Segars J.H., Parrott E.C., Nagel J.D., Guo X.C., Gao X., Birnbaum L.S., Pinn V.W., Dixon D. (2014). Proceedings from the third national institutes of health international congress on advances in uterine leiomyoma research: Comprehensive review, conference summary and future recommendations. Hum. Reprod. Update.

[4] Shtykalova S.V., Egorova A.A., Maretina M.A., Freund S.A., Baranov V.S., Kiselev A.V. (2021). Molecular Genetic Basis and Prospects of Gene Therapy of Uterine Leiomyoma. Russ. J. Genet..

[5] Nadine Markowski D., Tadayyon M., Bartnitzke S., Belge G., Maria Helmke B., Bullerdiek J. (2014). Cell cultures in uterine leiomyomas: Rapid disappearance of cells carrying MED12 mutations. Genes Chromosomes Cancer.

[6] Holzmann C., Markowski D.N., Bartnitzke S., Koczan D., Helmke B.M., Bullerdiek J. (2015). A rare coincidence of different types of driver mutations among uterine leiomyomas (UL). Mol. Cytogenet..

[7] Bloch J., Holzmann C., Koczan D., Helmke B.M., Bullerdiek J. (2017). Factors affecting the loss of MED12-mutated leiomyoma cells during in vitro growth. Oncotarget.

[8] Klemke M., Meyer A., Nezhad M.H., Bartnitzke S., Drieschner N., Frantzen C., Schmidt E.H., Belge G., Bullerdiek J. (2009). Overexpression of HMGA2 in uterine leiomyomas points to its general role for the pathogenesis of the disease. Genes Chromosomes Cancer.

[9] Wang J., Ohara N., Takekida S., Xu Q., Maruo T. (2005). Comparative effects of heparin-binding epidermal growth factor-like growth factor on the growth of cultured human uterine leiomyoma cells and myometrial cells. Hum. Reprod..

[10] Salas A., López J., Reyes R., Évora C., de Oca F.M., Báez D., Delgado A., Almeida T.A. (2020). Organotypic culture as a research and preclinical model to study uterine leiomyomas. Sci. Rep..

[11] Osinovskaya N.S., Malysheva O.V., Shved N.Y., Ivashchenko T.E., Sultanov I.Y., Efimova O.A., Yarmolinskaya M.I., Bezhenar V.F., Baranov V.S. (2016). Frequency and Spectrum of MED12 Exon 2 Mutations in Multiple Versus Solitary Uterine Leiomyomas From Russian Patients. Int. J. Gynecol. Pathol..

[12] Dzhemlikhanova L.K., Efimova O.A., Osinovskaya N.S., Parfenyev S.E., Niauri D.A., Sultanov I.Y., Malysheva O.V., Pendina A.A., Shved N.Y., Ivashchenko T.E. (2017). Catechol-O-methyltransferase Val158Met polymorphism is associated with increased risk of multiple uterine leiomyomas either positive or negative for MED12 exon 2 mutations. J. Clin. Pathol..

[13] Islam M.S., Protic O., Ciavattini A., Giannubilo S.R., Tranquilli A.L., Catherino W.H., Castellucci M., Ciarmela P. (2014). Tranilast, an orally active antiallergic compound, inhibits extracellular matrix production in human uterine leiomyoma and myometrial cells. Fertil. Steril..

[14] Moore A.B., Yu L., Swartz C.D., Zheng X., Wang L., Castro L., Kissling G.E., Walmer D.K., Robboy S.J., Dixon D. (2010). Human uterine leiomyoma-derived fibroblasts stimulate uterine leiomyoma cell proliferation and collagen type I production, and activate RTKs and TGF beta receptor signaling in coculture. Cell Commun. Signal..

