# Supplementary material for: Development of Primary Monolayer Cell Model and Organotypic Model of Uterine Leiomyoma
Source: Methods Protoc. 2022 Feb 6;5(1):16. doi: 10.3390/mps5010016 (PMC8875914; doi:10.3390/mps5010016)
Supplement: Supplementary file 1 [file mps-05-00016-s001.zip › mps-1471064-supplementary.pdf]

Supporting Information for the Article

Development of primary monolayer cell model and organotypic model of uterine leiomyoma

Natalia Shved[1], Anna Egorova[1], Natalia Osinovskaya[1] and Anton Kiselev[1,*]

[1] Department of Genomic Medicine, D.O. Ott Research Institute of Obstetrics, Gynecology and Reproductology, Mendeleevskaya line 3, 199034 Saint-Petersburg, Russia; natashved@mail.ru (N.S.), egorova_anna@yahoo.com (A.E.), natosinovskaya@mail.ru(N.O.)

* Correspondence: ankiselev@yahoo.co.uk; Tel.: +7-812-328-9809 (A.V.)

Contents

Figure S1. Detection of heterozygous mutation c.131G>A in MED12 gene in the same primary UL cell line at passages p0 and p1 cultured with AmnioMAX™ C-100 or DMEM F-12 medium……..3

Figure S2. Histological characterization of the organotypic model. Hematoxyline-eosine stained cryosections of UL fragments after 7, 14 and 21days of culture (magnification ×200)………...4

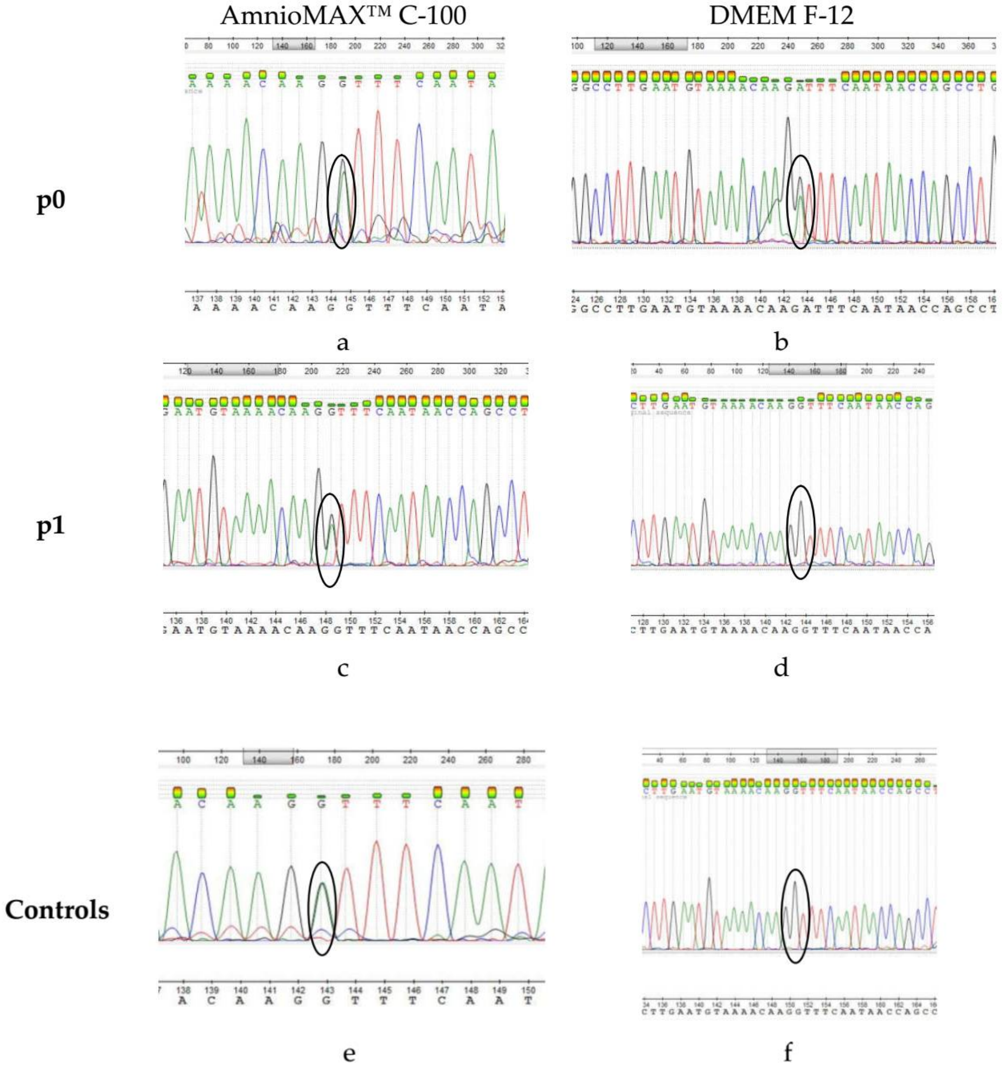

Figure S1. Detection of heterozygous mutation c.131G>A in *MED12* gene in the same primary UL cell line at passages p0 (**a, b**) and p1(**c, d**) cultured with AmnioMAX™ C-100 (**a, c**) or DMEM F-12 medium (**b, d**); controls – native myomatous node (**e**) and surrounding myometrium (**f**).

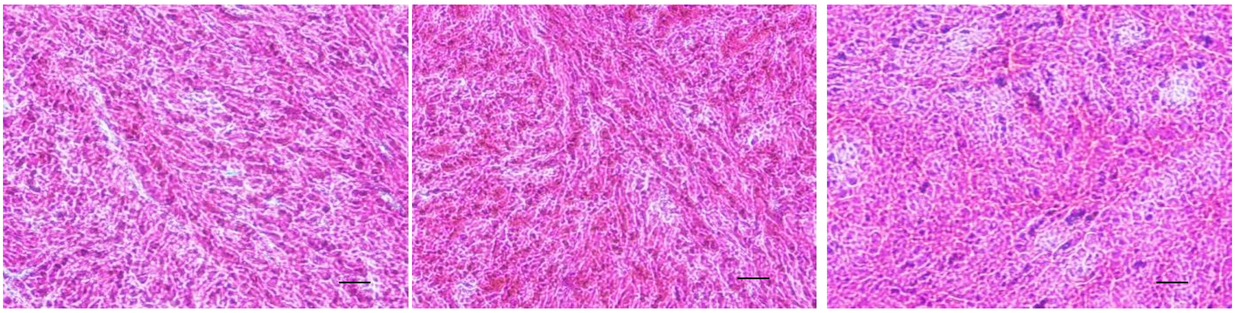

a b c

Figure S2. Histological characterization of the organotypic model. Hematoxyline-eosine stained cryosections of UL fragments after 7 (**a**), 14 (**b**) and 21(**c**) days of culture (magnification ×200; scale bar is 100 μm).
